# Supplementary material for: Global data-driven prediction of fire activity
Source: Nat Commun. 2025 Apr 1;16:2918. doi: 10.1038/s41467-025-58097-7 (PMC11962136; doi:10.1038/s41467-025-58097-7)
Supplement: Supplementary file 1 — Supplementary Information [file 41467_2025_58097_MOESM1_ESM.pdf]

Supplementary information associated to the manuscript 'Global data-driven prediction of fire activity'

We provide two additional information pieces. Figure S1 showing the IPCC AR6 regions referenced in Figure 1. Table S1 shows a table of the absolute values for the skill-scores depicted in the spider diagram of Figure 1 of the manuscript.

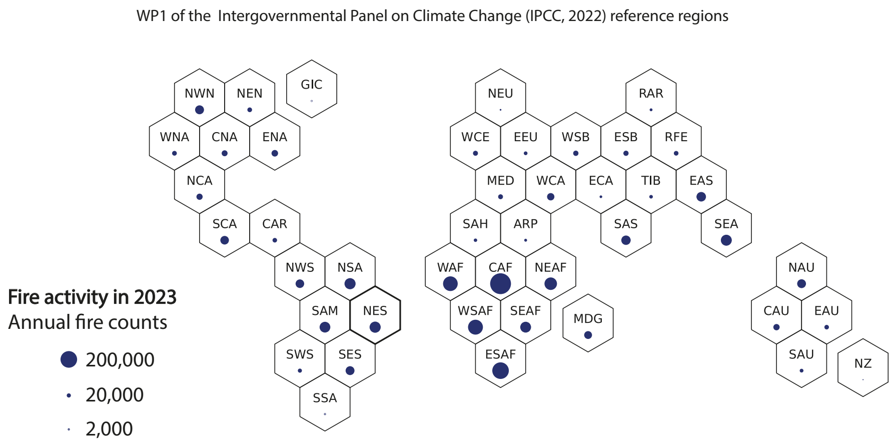

Fig. S1: Total fire activity recorded in 2023 on the hexagonal word representation of WP1 of the Intergovernmental Panel on Climate Change (IPCC, 2022). IPCC AR6 WGI reference regions are: **North America**: NWN (North-Western North America, NEN (North-Eastern North America), WNA (Western North America), CNA (Central North America), ENA (Eastern North America), **Central America**: NCA (Northern Central America), SCA (Southern Central America), CAR (Caribbean), **South America**: NWS (North-Western South America), NSA (Northern South America), NES (North-Eastern South America), SAM (South American Monsoon), SWS (South-Western South America), SES (South-Eastern South America), SSA (Southern South America), **Europe**: GIC (Greenland/Iceland), NEU (Northern Europe), WCE (Western and Central Europe), EEU (Eastern Europe), MED (Mediterranean), **Africa**: MED (Mediterranean), SAH (Sahara), WAF (Western Africa), CAF (Central Africa), NEAF (North Eastern Africa), SEAF (South Eastern Africa), WSAF (West Southern Africa), ESAF (East Southern Africa), MDG (Madagascar), **Asia**: RAR (Russian Arctic), WSB (West Siberia), ESB (East Siberia), RFE (Russian Far East), WCA (West Central Asia), ECA (East Central Asia), TIB (Tibetan Plateau), EAS (East Asia), ARP (Arabian Peninsula), SAS (South Asia), SEA (South East Asia), **Australasia**: NAU (Northern Australia), CAU (Central Australia), EAU (Eastern Australia), SAU (Southern Australia), NZ (New Zealand), Small Islands: CAR (Caribbean), PAC (Pacific Small Islands)

	**N.W.North America**				
	Correlation	Brier	Logloss	ROC	ECE
**PoF-Weather**	0.066	5.13E-04	0.0035	0.947	1.97E-04
**PoF-Fuel**	0.017	5.16E-04	0.0040	0.884	2.71E-04
**PoF-Ignition**	0.006	5.15E-04	0.0046	0.737	3.52E-04
**PoF-Fuel/Weather**	0.061	5.13E-04	0.0035	0.958	2.91E-04
**PoF-Ignition/Weather**	0.052	5.14E-04	0.0039	0.946	4.04E-04
**PoF-Ignition/Fuel**	0.014	5.15E-04	0.0043	0.887	3.93E-04
**PoF**	0.059	5.14E-04	0.0036	0.957	3.70E-04
	**N.South America**				
	Correlation	Brier	Logloss	ROC	ECE
**PoF-Weather**	0.065	2.80E-03	0.0188	0.818	3.49E-04
**PoF-Fuel**	0.102	2.78E-03	0.0178	0.871	7.45E-04
**PoF-Ignition**	0.087	2.79E-03	0.0173	0.831	8.58E-04
**PoF-Fuel/Weather**	0.119	2.77E-03	0.0171	0.896	2.61E-04
**PoF-Ignition/Weather**	0.106	2.78E-03	0.0175	0.879	1.11E-04
**PoF-Ignition/Fuel**	0.112	2.77E-03	0.0172	0.891	6.39E-04
**PoF**	0.128	2.76E-03	0.0168	0.905	2.27E-04
	**South American Monsoon**				
	Correlation	Brier	Logloss	ROC	ECE
**PoF-Weather**	0.038	4.45E-03	0.0277	0.703	1.83E-03
**PoF-Fuel**	0.028	4.64E-03	0.0281	0.746	1.56E-03
**PoF-Ignition**	0.033	4.44E-03	0.0286	0.639	1.23E-03
**PoF-Fuel/Weather**	0.076	4.48E-03	0.0265	0.809	2.56E-03
**PoF-Ignition/Weather**	0.081	4.42E-03	0.0260	0.798	6.97E-04
**PoF-Ignition/Fuel**	0.060	4.45E-03	0.0268	0.769	1.15E-03
**PoF**	0.088	4.43E-03	0.0257	0.82	1.54E-03
	**Mediterranean**				
	Correlation	Brier	Logloss	ROC	ECE
**PoF-Weather**	0.012	3.62E-04	0.0046	0.771	1.63E-03
**PoF-Fuel**	0.016	3.43E-04	0.0049	0.753	2.02E-04
**PoF-Ignition**	0.012	3.45E-04	0.0030	0.812	3.99E-04
**PoF-Fuel/Weather**	0.017	3.45E-04	0.0049	0.773	1.61E-04
**PoF-Ignition/Weather**	0.015	3.46E-04	0.0036	0.828	4.65E-04
**PoF-Ignition/Fuel**	0.018	3.43E-04	0.0048	0.762	1.63E-04
**PoF**	0.022	3.43E-04	0.0049	0.774	1.48E-04
	**Sahara**				
	Correlation	Brier	Logloss	ROC	ECE
**PoF-Weather**	0.006	1.58E-04	0.0044	0.634	3.16E-03
**PoF-Fuel**	0.027	1.28E-04	0.0031	0.688	7.47E-05
**PoF-Ignition**	0.029	1.30E-04	0.0014	0.763	2.97E-04
**PoF-Fuel/Weather**	0.022	1.29E-04	0.0031	0.688	7.11E-05
**PoF-Ignition/Weather**	0.022	1.33E-04	0.0018	0.754	3.79E-04
**PoF-Ignition/Fuel**	0.027	1.29E-04	0.0031	0.689	6.72E-05
**PoF**	0.035	1.28E-04	0.0030	0.689	6.37E-05
	**Western Africa**				
	Correlation	Brier	Logloss	ROC	ECE
**PoF-Weather**	0.115	6.31E-03	0.0356	0.839	1.22E-03
**PoF-Fuel**	0.138	6.27E-03	0.0340	0.877	1.91E-03
**PoF-Ignition**	0.114	6.31E-03	0.0340	0.832	1.41E-03
**PoF-Fuel/Weather**	0.161	6.23E-03	0.0330	0.892	1.07E-03
**PoF-Ignition/Weather**	0.156	6.24E-03	0.0336	0.877	6.57E-04
**PoF-Ignition/Fuel**	0.144	6.26E-03	0.0335	0.884	1.46E-03
**PoF**	0.164	6.23E-03	0.0328	0.895	1.10E-03
	**S.E.Asia**				
	Correlation	Brier	Logloss	ROC	ECE
**PoF-Weather**	0.127	1.09E-03	0.0081	0.933	6.16E-04
**PoF-Fuel**	0.104	1.09E-03	0.0081	0.926	3.59E-04
**PoF-Ignition**	0.074	1.10E-03	0.0070	0.918	1.70E-04
**PoF-Fuel/Weather**	0.149	1.08E-03	0.0079	0.935	3.26E-04
**PoF-Ignition/Weather**	0.137	1.09E-03	0.0078	0.935	3.76E-04
**PoF-Ignition/Fuel**	0.121	1.09E-03	0.0079	0.932	3.93E-04
**PoF**	0.156	1.08E-03	0.0078	0.936	3.05E-04
	**E.Australia**				
	Correlation	Brier	Logloss	ROC	ECE
**PoF-Weather**	0.022	1.51E-03	0.0117	0.737	1.73E-03
**PoF-Fuel**	0.029	1.55E-03	0.0125	0.761	3.52E-03
**PoF-Ignition**	0.055	1.49E-03	0.0108	0.735	5.49E-04
**PoF-Fuel/Weather**	0.039	1.51E-03	0.0113	0.793	2.01E-03
**PoF-Ignition/Weather**	0.057	1.49E-03	0.0107	0.775	4.21E-04
**PoF-Ignition/Fuel**	0.043	1.50E-03	0.0112	0.766	1.37E-03
**PoF**	0.053	1.49E-03	0.0106	0.798	7.67E-04
	**Global**				
	Correlation	Brier	Logloss	ROC	ECE
**PoF-Weather**	0.085	1.54E-03	0.0101	0.894	1.71E-04
**PoF-Fuel**	0.136	1.53E-03	0.0098	0.915	1.27E-04
**PoF-Ignition**	0.103	1.54E-03	0.0095	0.893	9.74E-05
**PoF-Fuel/Weather**	0.171	1.51E-03	0.0094	0.935	1.39E-04
**PoF-Ignition/Weather**	0.151	1.52E-03	0.0092	0.93	4.89E-05
**PoF-Ignition/Fuel**	0.154	1.52E-03	0.0096	0.922	1.12E-04
**PoF**	0.181	1.50E-03	0.0092	0.938	4.32E-05

Table S1 Table of the absolute values for the skill-scores depicted in the spider diagram of Figure 1 of the manuscript)
